# The distribution pattern of *Halicephalobus gingivalis* in a horse is suggestive of a haematogenous spread of the nematode

**DOI:** 10.1186/s13028-014-0056-0

**Published:** 2014-09-19

**Authors:** Christina Henneke, Anna Jespersen, Stine Jacobsen, Martin K Nielsen, Fintan McEvoy, Henrik E Jensen

**Affiliations:** Department of Veterinary Disease Biology, University of Copenhagen, Ridebanevej 3, DK-1870 Frederiksberg C, Denmark; Department of Large Animal Sciences, University of Copenhagen, Højbakkegård Alle 5, DK-2630 Taastrup, Denmark; M. H. Gluck Equine Research Center, Department of Veterinary Science, University of Kentucky, Lexington, Kentucky USA; Department of Veterinary Clinical and Animal Sciences, University of Copenhagen, Dyrlægevej 16, DK-1870 Frederiksberg C, Denmark

**Keywords:** *Halicephalobus gingivalis*, Pyogranulomatous, Encephalitis, Meningitis

## Abstract

The majority of *Halicephalobus gingivalis*-infections in horses have been fatal and are usually not diagnosed before necropsy. Therefore, knowledge about the nematode and the pathogenesis of infection in horses is limited. This has resulted in an on-going discussion about the port of entry and subsequent dissemination of *H. gingivalis* within the host. The present case of *H. gingivalis*-infection in a horse was diagnosed ante mortem. Post mortem findings, the distribution pattern of *H. gingivalis* nematodes in the brain, a high prevalence of inflammation in close relation to blood vessels, and the presence of the nematode in multiple organs with a disseminated pattern of distribution strongly suggested a haematogenous spread of the nematode in the horse.

## Background

*Halicephalobus gingivalis* is a free-living nematode belonging to the order Rhabditida and family Panagrolaimidae [1]. *H. gingivalis* is a facultative parasite of humans and horses and more than five cases in humans and 65 cases in horses have been reported [2] since the infection was described for the first time by Stefanski in 1954 [3]. The majority of infections have been fatal and in most cases the diagnosis was stated post mortem. In horses, *H. gingivalis* mainly infects the brain, kidneys, mandible and maxillary bones and the eyes where it causes a granulomatous inflammation [4-6]. In single cases, the nematode has been found histologically in the heart, blood vessels, testicles, femur, lymph nodes, parathyroid glands and preputium [2,5,7]. There is an on-going discussion about the pathogenesis of the nematode, especially concerning the spreading following penetration into the body. Different theories including haematogenous [4-6] and lymphogenic [4] spread and tissue migration [8,9] have been proposed.

An equine case of *H. gingivalis* infection was recently diagnosed at the University of Copenhagen, and lesions in various organs were strongly supportive of a haematogenous dissemination of the nematode.

## Case presentation

In July 2012, an 11-year-old Icelandic horse stallion with a history of fever and unilateral facial swelling was admitted to the University Hospital for Large Animals. Clinically, the horse had bilaterally enlarged mandibular lymph nodes and a bony proliferation above the maxillary sinus. Bilateral opening of the maxillary sinuses was performed with a bone flap technique at standing surgery [10]. Large amounts of granulation tissue like masses filled most of the caudal and rostral parts of the left maxillary sinus. The masses were debrided extensively and tissue samples fixed in 10% neutral buffered formalin, embedded in paraffin wax and sectioned at 4 μm. Sections were stained with haematoxylin and eosin (HE). Histologically, large numbers of concentric pyogranulomatous foci with centrally placed *H. gingivalis*-like nematodes were observed. Moreover, tissues samples were examined for parasites by the Baermann method [11] by which several adult *H. gingivalis* nematodes were identified based on morphological appearance [1]. Only female specimens were identified.

The horse was treated with a second sinus surgery, where the flap was reopened and the sinuses packed with gauze soaked in ivermectin. Postoperatively, sinuses were flushed with ivermectin once a day through trephination holes. Additionally, the horse received fenbendazole at twice the recommended dosage (15 mg/kg) once a day for 5 consecutive days and auxiliary therapy with penicillin (22 000 IU/kg), flunixin meglumine (1.1 mg/kg), dexamethasone (0.1 mg/kg), omeprazole (1 mg/kg), as well as fluid and pain medication as needed.

The horse developed severe neurological signs (ataxia and blindness), which progressed over the next week. Due to the lack of response to treatment and the grave prognosis, the horse was euthanized and a post mortem computed tomography (CT) scanning of the head only revealed lesions within the sinuses. The horse was subjected to a complete necropsy, and following removal of the brain, the head was sagitally sectioned at different levels. The observed granulation tissue-like masses in the left rostral and caudal maxillary sinuses had caused a marked compression of the nasal cavity.

In addition to the sinus lesions, macroscopic renal lesions were found. These were seen as multiple disseminated, yellow-grey nodules with a diameter of 1.0 to 1.5 cm protruding from the surface of both kidneys.

**Figure 1:**
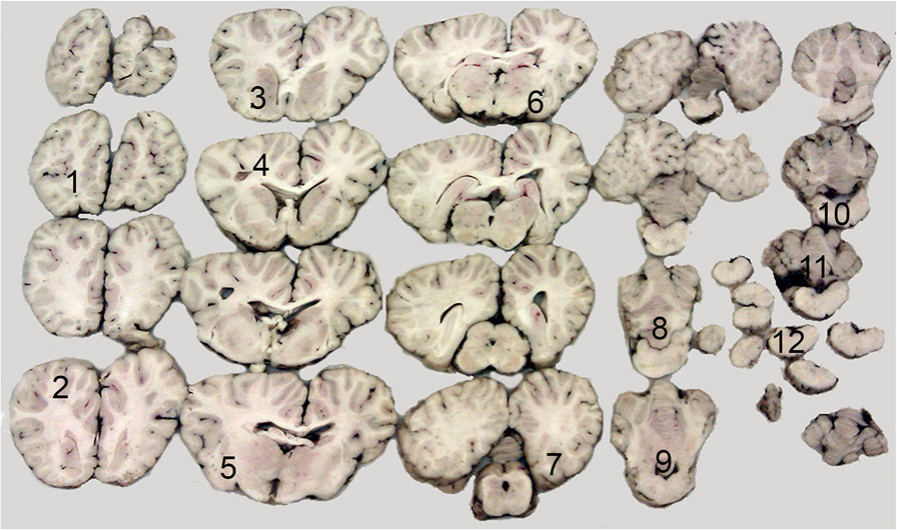
**Brain section of equine brain with the sample numbers (1–12) taken for histology.** In Figure 2 the degree of inflammation in relation to the 12 samples/areas of the brain is illustrated.

**Figure 2:**
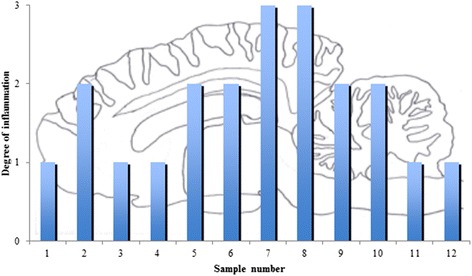
**The degree of inflammation (mild = 1, moderate = 2, and severe = 3) in relation to the 12 sample sites, see Figure** 1 **.**

**Figure 3:**
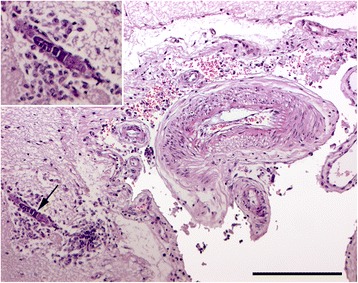
**In sample number 4 from the left hemisphere, a mature female nematode with dorso flexed ovary (→) is located in the brain tissue (inserted) just beneath the meninges.** The nematode is accompanied by macrophages in the brain tissue and a mild infiltration of mononuclear cells in the leptomeninges. HE. Bar = 100 μm.

For histology, the brain was sampled *in toto* together with tissue specimens from the sinuses, kidneys, lungs, liver, eyes, and spleen. The samples were processed as described above and sections were stained with HE. As illustrated in Figure 1, a total of 12 samples were taken from the brain, and in all sections some degree of inflammation was seen in the meninges and brain parenchyma. Lesions were mainly observed in the midbrain (Figure 2). In the brain parenchyma, lesions were typically localized just beneath the meningeal lining or in relation to blood vessels (Figure 3). In the meninges, mild to severe inflammation was observed with infiltration of macrophages, multinucleate giant cells, lymphocytes as well as few plasma cells and eosinophils. In some areas, infiltration with neutrophils was present as well. The inflammatory reactions were mainly localized in relation to blood vessels but more diffuse infiltrations were also seen. Similar lesions were present in the brain tissue together with perivascular cuffs. In relation to the inflammatory reactions of the meninges and brain tissue, graded as mild, moderate and severe = 1, 2 and 3 (see Figure 2), numerous nematodes of all stages from eggs to adults, with an identical morphology to those present in the sinusoidal tissue masses, were present.

In both kidneys, a high density of parasites with the same characteristics as the nematodes in the meninges, brain and sinuses were found. The inflammatory reaction of the kidneys was similar to that in the sinuses, i.e. formation of concentric foci with nematodes localized centrally and surrounded by epithelioid cells, macrophages, multinucleated giant cells, lymphocytes, and occasionally eosinophils.

## Conclusions

The present case is comparable to other cases of equine *H. gingivalis*-infection [5], and the nematode was identified both in the histological samples of different organs and with subsequent isolation and identification. Knowledge about the nematode and infection of horses is limited due to the relative low number of reported cases and presumably because the infection is rarely diagnosed ante mortem. Moreover, reported clinical manifestations in horses with *H. gingivalis-*infections are highly variable, complicating any possible conclusions regarding the port of entry and subsequent dissemination of the parasite within the host. Consequently, this has given rise to an on-going discussion about this subject. However, during recent years the infection has been diagnosed in countries worldwide [2], and the growing number of reported cases has created a clearer picture of the route of infection and the distribution pattern of nematodes in horses. Several studies suggest that penetration of mucosal membranes or the skin is the most likely port of entry [7,8,12-14].

Nematodes are often found in various parts of the brain and meninges, and the high occurrence of neurological manifestations in horses with *H. gingivalis*-infection is probably caused by a high affinity for the meninges and brain tissue. This was confirmed in our study where inflammation and nematodes were found in a pattern of distribution as seen in animal models focusing on the localization and distribution of brain abscesses following challenge with *Staphylococcus aureus* [15]. The pattern of distribution of meningeal and brain lesions together with a high prevalence of inflammation and presence of nematodes in relation to blood vessels strongly suggest, that *H. gingivalis* was spread haematogenously to the brain and meninges in this case. This pathogenesis is supported by others, who observed the nematode close to blood vessels, and occasionally in the heart [13], and within the wall of blood vessels [13,14]. Although *H. gingivalis* has been isolated from urine and semen [16], it remains to be found in blood samples. A haematogenous mode of dissemination was also supported by the presence of bilateral and disseminated lesions in the kidneys, which have also frequently been reported by others [2,4,14,16].

The fact that nematodes occasionally were found in the brain parenchyma just beneath infected meningeal tissue indicates that some degree of local migration may occur. Histologically, all lesions were chronic and contained a high number of reproducing mature nematodes, which might give rise to the formation of new focal infection sites, just not visible macroscopically. *H. gingivalis* does not always cause gross lesions. It is occasionally found in other organs, e.g. in a macroscopically normal parathyroid gland of a horse with a suspected diagnosis of dystrophia fibrosa [2]. Moreover, as observed in the present case, the application of CT scan is often not helpful in order to disclose lesions not being apparent at gross inspection [17].
